# Behavioral model-guided nutritional counseling could improve the dietary practice and nutritional status of elders in Ethiopia: a quasi-experimental study

**DOI:** 10.1186/s12877-023-04433-9

**Published:** 2023-11-20

**Authors:** Ahmed Muhye Seid, Netsanet Fentahun Babbel

**Affiliations:** 1 School of Public Health, College of Medicine and Health Sciences, Bahir Dar University, Bahir Dar, Ethiopia; 2 Department of Public Health, College of Medicine and Health Sciences, Dire Dawa University, Dire Dawa, Ethiopia; 3 Department of Nutrition and Dietetics, School of Public Health, College of Medicine and Health Sciences, Bahir Dar University, Bahir Dar, Ethiopia

**Keywords:** Nutrition, Geriatrics, Nutritional status, Malnutrition, Nutrition counselling, Ethiopia

## Abstract

**Background:**

Physiological, pathological, and socioeconomic changes occurring in older people negatively influence food intake, utilization, nutritional status, and health. These problems are deeply rooted in low socio-economic settings and could partly be addressed through systematic behavioral change approaches. Hence, this study was to evaluate the effect of behavioral model-guided nutritional counseling on the dietary intake and nutritional status of elders.

**Methods:**

A one-armed pre- and post-test quasi-experimental design was conducted on 293 community-dwelling older adults aged above 60 years from January to May 2022. A health education tool was developed and validated using health beliefs and the theory of behavioral change. The sessions were delivered by trained nurses through home-to-home visits every week lasting 45–60 min for up to two months. Data on nutritional knowledge, dietary intake, and body weight were captured using standardized questionnaires and measurements. The primary outcome was captured using the validated mini-nutritional assessment (MNA) tool and classified accordingly. The data was analyzed using Stata software, where it is presented in tables, graphs, and summary statistics. A paired t-test and the p-value were used to identify statistically significant effects of the intervention.

**Results:**

A total of 263 elders were involved in the experiment, and modeled nutritional counseling significantly improved the knowledge score from 7.58 (± 1.05) to 11.6 (± 1.37) (*P* < 0.001) at the pre- and post-intervention periods. A significant improvement has been shown in the consumption of dairy products, fruits, and animal-source foods and, importantly, in the mean dietary diversity score (*p* < 0.001). As a result, the burden of malnutrition was significantly lower in the post-intervention period (9.6%: 7.9–11.3) compared to baseline (12.5%: 11.4–13.8). There is a significant increase in the mini-nutrition assessment score (MD = 0.30; *p* = 0.007). The mean body weight and the body mass index did not change significantly after the intervention (*p* > 0.05).

**Conclusion:**

Targeted behavioral model-guided nutritional counseling could help promote perception, diversify dietary consumption, and reduce the risk of undernutrition among elders. Particular attention to older people with the use of participant-centered nutritional behavioral change interventions coupled with livelihood support could help reduce undernutrition among older people.

**Trial registration:**

Clinical Trial Registration-URL: www.clinicaltrials.gov, identifier number: NCT04746664, first released 10/02/2021.

## Background

In Africa, however, there is a shortage of information as well as epidemiological data on the nutritional status and intervention outcomes of the aging population [1, 2]. Furthermore, nutritional interventions in sub-Saharan Africa do not target older people [3], and, equally, social support from families and communities has declined largely due to widespread poverty, urbanization, population displacements, and globalization [2]. At the same time, demographic growth and population aging are occurring in Ethiopia in the absence of adequate economic and infrastructure systems [1]. Globally, the size of the older population is rising alarmingly, and the population will double by 2050 [4].

Physiological, pathological, and socioeconomic changes that occur in old age could influence their dietary intake, efficiency of nutrient utilization, and nutritional status [5, 6]. A study showed that 51.7% and 15.7% were found to be at risk of malnutrition and malnourished, respectively [7]. In Ethiopia, about 21% of older people are undernourished, which is associated with depression and low socioeconomic class [8]. Available studies in the country reported a 10.3% [9] to 28.3% [10] prevalence of malnutrition among the elderly. Elderly people are one of the most neglected population segments, yet malnutrition significantly increases the risks for morbidity and mortality [11, 12]. Hence, promoting optimal nutritional status is a key to improving longevity and quality of life among elders. Undernutrition among the elderly increases frailty, reduces cognitive performance, and predisposes them to age related degenerative disease [13]. This could be addressed through targeted behavioral interventions to fill knowledge gaps and improve the quality of positive behaviors [11, 14]. These can be addressed by behavioral change-modeled nutritional counseling [15].

Malnutrition in old people is basically addressed through periodic assessment and individualized treatment plans [16]. Moreover, individualized dietary counseling, a method by which people are guided and helped to put healthy foods and lifestyles into practice, is more effective than similar advice given for all old people, as they greatly vary in terms of biological age, disease conditions, functional frailty, and nutritional status [17]. According to studies, nutritional counseling is a crucial tool for maintaining good health and is a recognized component of the treatment of eating disorders [17, 18]. A recent review study showed nutritional counseling for malnourished older people had a positive impact on their body composition, weight, and grip strength [19]. Furthermore, theoretical or behavioral models such as the health belief model (HBM) and the theory of planned behavior (TPB) guide nutritional interventions that are more effective in encouraging behavior change than those that are not [20, 21]. The Academy of Nutrition and Dietetics also advises health practitioners to employ a theoretical strategy as described in the Nutrition Care Process (NCP) [22].

Countries like Ethiopia are adopting various strategies with the inclusion of multifaceted approaches to address the nutritional problems of the elderly, though it is in the early stages [23, 24]. To support such interventions and initiatives, it is crucial to have context-specific evidence on potential intervention approaches to prevent undernutrition [24]. Such huge burden of malnutrition can impact risks of morbidity, mortality, rapid progression of aging and reduced quality of life. However, there is lack of intervention trials evaluating their impacts at scale. Yet, there is a lack of research examining the advantages of tailored nutritional counseling in the case of Ethiopia, which can be extrapolated for other similar settings as well. Thus, the current study would be novel in its implementation of modeled nutritional counseling for dietary diversity and nutritional status among elders.

## Materials and methods

### Study design and period

A single-group pretest–posttest quasi-experimental design was conducted in Bahir Dar City, Northwestern Ethiopia. Bahir Dar is the capital city of the Amhara regional state, and it is a well urbanized city located in the northwest part of Ethiopia. Since there is no usual nutritional care system for old people in Ethiopia, it was unethical to include a control group that would have received no dietetic intervention, as nutritional care should be provided to those in need for a better outcome [25]. The baseline survey was conducted from January 28 to February 8, 2022; the nutrition counseling intervention was implemented from February 14 to March 15, 2022; and the end-line survey was conducted from May 16 to 26, 2022 after two months. We decided to have a reasonably optimal time span to affirm the response from the intervention. It is evident that intervention over one month might not lead to full achievement of the outcome of interest. However, behavioral intervention usually has stages and some of them could be captured within short period of time and sometimes longer follow-up could lead to recall bias and the intervention effects tend to be confounded. Hence, considering the aforementioned reasons and the practical feasibility of the study, we reasonably determined the follow-up period to be two months.

### Study population

This study included community-dwelling old people aged 60 and above who lived at least six months in the selected kebeles (the smallest administrative unit in Ethiopia) and desired to live three months in the area. We excluded old people who were on therapeutic dietary supplements and those who were seriously ill during the intervention period making them weak and unable to communicate easily like acute illnesses affecting their daily routine, severe acute febrile illness and making them unable to comprehend the counseling sessions. The findings of this study would be informative for elders in Bahir Dar city and Amhara region at large.

### Sample size and sampling procedure

The sample size for this study was estimated using the sample size for the before-after study or paired T-test [26], which is validated and applicable in many settings. Hence, we used a 95% confidence level, 80% power, 20% effect size, and one arbitrary standard deviation of the change in the outcome since no previous study was found in Ethiopia. The final sample size was 323 after accounting for 1.5 design effects and 10% lost to follow-up.

A multi-stage cluster sampling technique was used to address the study participants. Since Bahir Dar City Administration has six sub-cities, and two of them (Belay Zeleke and Gish-Abay) were used for previous studies, two other sub-cities, Tana and Dagmawi Minilik, were selected using a simple random sampling method in the first stage. In the second stage, two clusters of Kebeles, the smallest administrative unit in Ethiopia, were selected from each sub-city using the lottery technique. Lastly, all eligible old age people found in the selected kebeles were contacted in a home-to-home visit. If the targeted household was closed, a second visit was conducted. If the household was not open again, it was left unattended and taken as a non-respondent. When there was more than one eligible person in a household, one participant was chosen by a random lottery method.

### Intervention approach

Individual-based nutritional counseling guided by a combination of the HBM and TPB was implemented. The counseling package was adapted from previous studies on older people in other countries [27, 28]. The intervention program was a personalized intervention involving 45 to 60-min face-to-face sessions conducted home-to-home once per week for a one-month period and weekly individual home-to-home visit follow-up for two months. Eight trained nurses provided the nutritional counseling, and a convenient day and time were selected in discussion from all seven days of the week. The main message was written on flyers in the local language (Amharic) and given to each study participant as a reminder to lead a healthy lifestyle. Any family member or neighbor was asked to read the flyer to the elderly and/or other family members on behalf of those who could not read.

The objective of the intervention was not to completely change the participants’ or family’s food habits, but to correct possible shortfalls in their diet based on the food items that they are familiar with and that are already part of their daily diet. The main intention was to increase awareness of the importance of a balanced diet intake; improve the regularity of food intake (three major meals as breakfast, lunch, and dinner) with the addition of snacks between them; increase consumption of food varieties, especially fruits and vegetables, whole grains, and protein-rich foods without avoiding any specific foods except in medical cases; drink at least eight glasses of water; consume fewer foods with sodium, added sugar, saturated fat, or refined grains; and control the amount of noncaloric beverages consumed per day.

### Intervention fidelity

A checklist of criteria was constructed to evaluate the fidelity of the intervention based on the best practice recommendations of the National Institutes of Health Behavior Change Consortium (NIHBCC) framework [29]. The checklist tried to evaluate the intervention design, training of counselors, counseling delivery, receipt of counseling, and enactment of knowledge and skills gained from the intervention.

The intervention design was supported by theoretical models. To standardize the procedure, each study participant had the same number and frequency of counseling sessions, and the minimum lengths of contacts were comparable. The counsellors were trained in groups using a standardized training manual, role plays, and simulations of consulting practice, followed by feedback to the provider using performance criteria. Moreover, a written pre-post-training test was utilized to evaluate the counsellors’ knowledge and skill acquisition. A minimum of 80% was accepted, and those who scored below this were given remedial training.

The principal investigator monitored the counseling delivery process. They coached and evaluated randomly selected consulting sessions for each counselor. The evaluator used a "yes/no" rating system to check items such as the use of the counseling guide, the provision of all the content, the duration and frequency of the counseling, and the ability to answer questions correctly. Feedback was given to the counselors after each assessment to improve the observed gaps. The homework tasks were set at the end of each session to reflect the discussions in that session, and the counselor reviewed them in the following lesson. The reasons for not completing homework were explored, as these reasons may indicate a lack of understanding or hinder the implementation of learning skills.

In addition, the intervention’s enactment or implementation of knowledge acquired from the intervention was assessed through participants’ self-report. The study participants were interviewed about the change in their dietary intake, meal frequency, and variety following the nutrition consultations. Besides, counselors recorded participant attendance and self-reported knowledge measures at the end of counseling session. Those old age people who did not attend all consultation sessions were considered to "not adhere to the guideline", while those who dropped out of the study were considered to be "lost to follow-up".

Furthermore, the participant satisfaction survey was taken after a week. Finally, at the end of three months, participants were questioned with eight open-ended questions created by the researchers to learn how they felt about the program in terms of its scope, fidelity, the length of the counseling sessions, and whether or not their personal expectations were realized.

### Data collection instruments and procedures

Data were collected in pre- and post-tests using structured, interviewer-administered questionnaires and anthropometric measurements. The wealth index was assessed based on household assets, household water sources, and latrine conditions adapted from the Ethiopian Demographic Health Survey (EDHS) [30]. Food access was examined through the Household Food Insecurity Access Scale (HFIAS) [31], which has been previously utilized in Ethiopian studies [32, 33]. If the aged person is living with others, household head females were the respondents for this part since they are culturally responsive to handling household food consumption [34].

The nutritional status was measured using a validated Mini Nutritional Assessment tool, while the dietary practices were evaluated using the 24-h dietary recall, meal frequency, and dietary diversity score methods. Furthermore, a true–false or “I do not know” questionnaire was utilized for general nutritional knowledge questions (GNKQ), and a five-point Likert scale ranging from 1 (strongly disagree) to 5 (strongly agree) was utilized to measure the HBM and TPB constructs.

Wealth scores were created to classify households as the poorest, middle, and richest [30]. Each respondent received a total score for food access that ranged from 0 to 27, with higher scores indicating greater food insecurity or inaccessibility and vice versa [31]. The 16 food items in 24 h dietary recall were merged into eight mutually exclusive food groups to find foods with similar nutritional profiles [35]. Consumption of each food group item was given one point, and a diet with at least four dietary diversity scores (DDS) was taken as nutritionally adequate or otherwise inadequate [35, 36] for the total number of foods consumed per day.

The nutritional status is given a maximum of 30 points and is classified as malnourished (< 17), at-risk (17–23.5), or well-nourished (≥ 24). For the 18 items of the GNKQ, each correct answer got one point, and each incorrect answer and “I do not know” got zero. The scoring was reversed for items that represent misinformation. The constructs of HBM and TPB were measured by the mean score of a five-point Likert scale. There were six BSc nurses and six urban health extension workers as data collectors, and a master nutritionist, together with the investigators, served as supervisors. Besides, another eight experienced BSc nurses were enrolled as intervention counselors.

### Data processing and analysis

The data were entered into EpiData version 3.1 [37] and exported to SPSS version 23 [38] for data cleaning and analysis. Descriptive statistics for categorical data were expressed in frequencies and percentages, whereas mean and standard deviation (SD) were used for continuous data. The effects of the intervention on outcomes were evaluated using a paired t-test. All assumptions were checked before each test, and a P-value of less than 0.05 was considered statistically significant.

### Data quality management

The study was registered at the Clinical Trial Registration [39] with identifier number NCT04746664 and the Transparent Reporting of Evaluation with Nonrandomized Designs (TREND) [40] guideline was used for the reporting of the results. Furthermore, both before and after intervention measurements were conducted using the same set of materials. Anthropometric guidelines and recommendations [41, 42] as well as COVID-19 precautions, were strictly followed. The data collectors and supervisors took two days of training on the study’s purpose and the utilization of data collection tools. Though randomization and masking were not carried out due to the nature of the study, participants, counselors, and data collectors were blinded to the study hypothesis.

### Ethical approval

This study was ethically approved by the Institutional Ethical Review Board (IERB) of Bahir Dar University, College of Medicine and Health Sciences (001/2021). Written informed consent was obtained from each study subject after explaining the study's aim and procedures in detail. When this is not applicable, consent is obtained from their legal guardians, family, or caregivers. Furthermore, official letters of cooperation obtained from the IERB were given to the Bahir Dar City Administrative Health Department and then to the respective offices of the selected study sub-cities and Kebeles (level four administrative division in Ethiopia) administrates. The confidentiality of the information was kept confidential with the investigator, and the data were captured without personal identifiers. This study was carried out in accordance with the Declaration of Helsinki's rules [43].

## Results

### Socioeconomic characteristics of the study participants

**Fig. 1:**
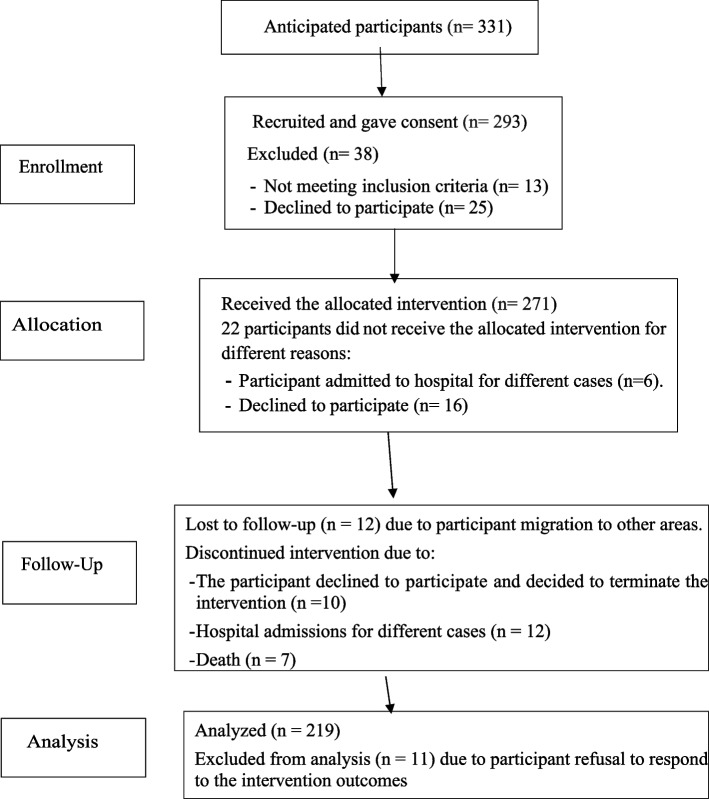
Recruitment procedure and flow chart of the study, Bahir Dar City, northern Ethiopia

There were 331 community-dwelling old people living in the selected cluster kebeles. A total of 293 people aged 60 years and older were recruited. Of them, 271 took the allocated intervention, but only 219 (80.8%) finished the intervention and were included in the statistical analysis. The remaining 13 old people were unable to meet the inclusion criteria; 62 declined to participate in different stages of the intervention; 18 were admitted to the hospitals for various reasons; and seven deaths were reported. The recruitment procedure and flow chart of the study are described in Fig. 1 and the figure legends.

Self-reported participants’ ages ranged from 60 to 98 years old at the baseline and from 60 to 92 years after the intervention. The mean age with standard deviation was 73.1 ± 7.3 before and 72.3 ± 6.8 years after the intervention, respectively. Three-fourths of respondents (75.3% before and 77.6% after) had access to food, whereas one-fourth (24.7% before and 22.4% after) had experienced mild to severe food insecurity in the four weeks before the survey (Table 1).

**Table 1:** Socioeconomic characteristics of the study participants, Bahir Dar City, northern Ethiopia

Variables	Categories	Pretest N (%) (*n* = 271)	Posttest N (%) (*n* = 219)
**Sex**	Female	148(54.6)	132(60.3)
	Male	123(46.4)	87(39.7)
**Age**	60–64	4(1.5)	4(1.8)
	65–69	78(28.8)	74(33.8)
	70–74	91(33.6)	68(31.1)
	75–79	42(15.5)	33(15.1)
	80–84	29(10.7)	23(10.5)
	≥ 85	27(10.0)	17(7.8)
**Religion**	Orthodox	190(70.1)	159(72.6)
	Islam	81(29.9)	60(27.4)
**Marital status**	Married	128(47.3)	106(48.4)
	Divorced	47(17.3)	43(19.6)
	Widowed	96(35.4)	70(32.0)
**Educational level**	Unable to read and write	144(53.1)	114(52.1)
	Can read and write	84(31.0)	72(32.9)
	Grade 1–8	36(13.3)	30(13.7)
	Grade 9 & above	7(2.6)	3(1.4)
**Current occupation**	Housewife	16(5.9)	12(5.5)
	Government employed	10(3.7)	7(3.2)
	Merchant	86(31.7)	74(33.8)
	Daily laborer	2(0.7)	2(0.9)
	No work/retired	157(57.9)	124(56.6)
**Live with**	Alone	56(20.7)	51(23.3)
	With others	215(79.3)	168(76.7)
**Household Wealth index**	Poorest	52(19.2)	42(19.2)
	Poor	17(6.3)	14(6.4)
	Middle	92(33.9)	72(32.9)
	Rich	52(19.2)	39(17.8)
	Richest	58(21.4)	52(23.7)
**Food insecurity**	Secured access	204(75.3)	170(77.6)
	Mild insecure access	60(22.1)	45(20.6)
	Moderate insecure access	2(0.7)	2(0.9)
	Severely food insecure	5(1.9)	2(0.9)

### Nutritional status of elderly

**Fig. 2:**
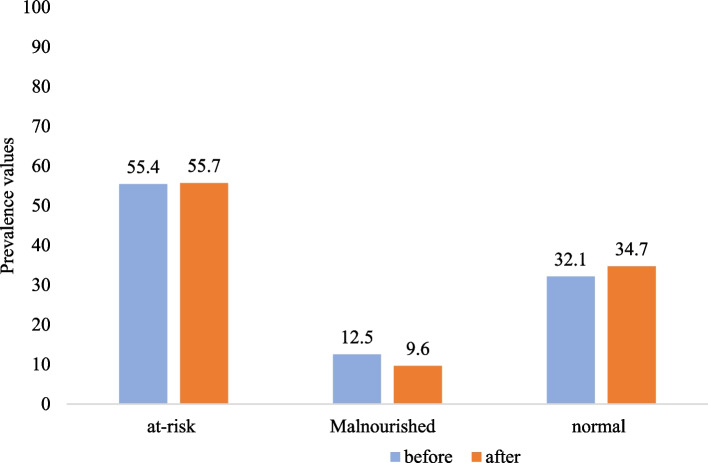
Nutritional status of the study participants before and after the nutritional counseling, Bahir Dar City, northern Ethiopia

The MNA tool revealed that more than half of the study participants were at risk of malnutrition before (55.4%) and after (55.7%) the nutritional counseling intervention. Only 34 (12.5%: 11.4–13.8) of them were malnourished before implementing the intervention, compared to 21(9.6%:7.9–11.3) in the second evaluation after the Program (Fig. 2).

Overall, the mean score of MNA increased from 21.55 ± 3.72 before the implementation of the intervention to 21.64 ± 4.12 immediately after the Program. The mean difference was statistically significant (x̄ = -0.30, t_218_ = -2.72, 95% CI: -0.52- (-0.83), *p* = 0.007). The mean body weight and BMI, however, did not change after the program's adoption. More than one third (37.3% and 37.9%) of the participants were underweight, while less than a quarter (22.5% and 20.6%) of them were overweight or obese before and after the program's implementation, respectively. The mean body weight was 58.2 ± 7.6 kg before and 57.9 ± 7.5 kg after the intervention.

### Nutritional knowledge of elderly

In the post-test, every study participant (*n* = 219) said they had gone over the counseling materials offered. However, 263(97%) of the study participants before the intervention and 204 (93.2%) after the intervention were aware that the quantity of food should change as people age. While only 6(2.2%) and 170 (77.6%) of them were aware that the quality of food should change as people aged before and after the counseling intervention, respectively. Besides, 162(74.0%) knew that old age people should eat more frequently than three times daily. Nevertheless, 35 (16.0%) and 28 (12.8%) of the study participants were still unaware that old age people should consume less fatty and salty food than adults, respectively.

The mean nutrition knowledge scores of the study participants considerably increased from 7.58 ± 1.05 before the implementation of the counseling intervention to 11.61 ± 1.37 immediately after the intervention. There was a significant average difference between pre- and post- test scores (*t*_218_ = -32.79, *p* < 0.001). Post-test scores were 3.98 points higher on average than pre-test scores (95% CI: -4.21-(-3.73)). Overall, 125(57.1%) of study participants after the counseling were found to have good knowledge, as opposed to 137(50.6%) before. In contrast, 134 (49.4%) and 94(42.9%) had scored poorly on knowledge tests conducted before and after the training, respectively.

### Constructs of the behavioral models

The mean score of all health belief models and theory of planned behavior constructs changed after the intervention. The mean score of the perceived susceptibility, severity, benefits, behavioral beliefs, cues to action, and intentions increased after the nutrition counseling compared to before the intervention. The result of the paired t-test also showed that there was a statistically significant difference (*P* < 0.05), except for the constructs of perceived severity, behavioral beliefs, and cues to action.

On the other hand, the mean score of perceived barriers, normative beliefs, and perceived self-efficacy decreased after the intervention, yet, the paired t-test result showed a statistically significant difference (*P* < 0.001) only for the perceived barriers (Table 2).

**Table 2:** Behavioral Model Constructs of Study Participants, Bahir Dar City, 2022

**Behavioral Model Constructs**	Pre-test (n1 = 271)		Post-test (n2 = 219)		*p*-value
	Mean	SD	Mean	SD	
Perceived susceptibility	8.61	1.40	8.69	1.64	< 0.001^*^
Perceived severity	15.07	1.43	15.13	1.50	0.318
Perceived benefits	19.04	1.86	19.78	1.80	< 0.001^*^
Perceived Barriers	34.14	1.50	33.99	1.59	< 0.001^*^
Behavioral beliefs	36.00	0.06	36.01	0.10	0.083
Normative Beliefs	10.63	0.93	10.60	0.92	0.318
Perceived Self-efficacy	8.64	1.98	8.53	1.98	0.18
Cues to action	7.62	0.30	8.83	0.34	0.14
Intentions	16.01	0.09	16.04	0.20	0.035^*^

^*^P-value is significant (*P* < 0.05) according to paired sample t-test

### Dietary practice of elderly

The majority of the respondents, 180(66.4%) and 152(69.4%) had regular meal practices before and after the intervention, respectively. An estimated 65.3% had three meals per day both before and after the intervention, while 92(33.9%) and 71(32.4%) of them had two meals per day before and after the intervention, respectively. However, none and only five (2.3%) of the respondents took the recommended five meals every day, respectively, before and after the intervention.

**Fig. 3:**
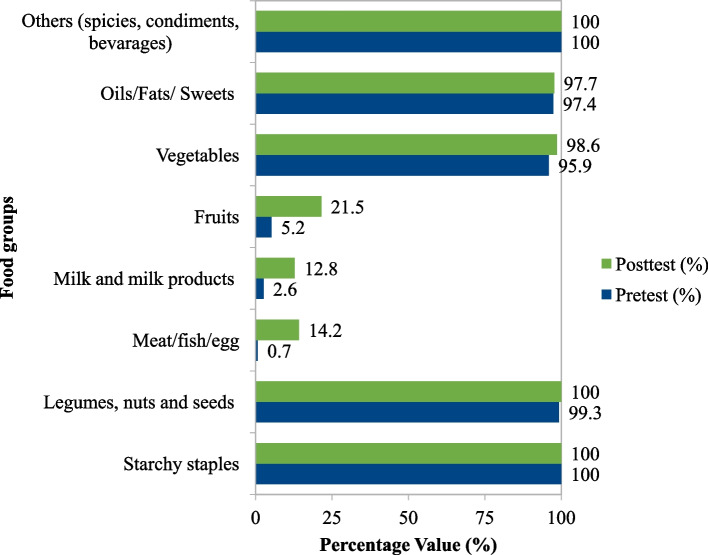
Different food groups consumed by study participants in Bahir Dar City, northern Ethiopia

The study participants consumed nearly identical dietary varieties before and after the intervention. Starchy staples, legumes, and spices were the most commonly consumed food groups, whereas animal products, fruits, and vegetables were rarely consumed before and after the intervention. Still, the study participants increased their consumption of dairy products (x̄ = -0.10, t_218_ = -4.19, 95% CI: -0.14-(-0.05), *p *< 0.001), meat/fish/egg (x̄ = -0.13, t_218_ = -5.77, 95% CI: -0.09-(-0.18), *p *< 0.001), and fruit (x̄ = -0.13, t_218_ = -5.77, 95% CI: -0.09-(-0.18), *p* < 0.001) after the counseling intervention. However, their changes in vegetable intake were not statistically significant (x̄ = -0.01, t_218_ = -1.0, 95% CI: -0.01-(-0.03), *p* = 0.32) (Fig. 3).

The dietary diversity score (DDS) increased from 5.00 ± 0.34 before the intervention to 5.37 ± 0.70 after the intervention, and the change was statistically significant (x̄ = -0.38, t_218_ = -7.17, 95% CI: -0.48-(-0.27), *p* < 0.001). Similarly, the proportion of participants having adequate DDS (having four or more DDS) increased from 254(93.73%) before to 209(95.43%) after the intervention.

## Discussion

This study was conducted to investigate the effect of behavioral model-guided nutritional counseling on the nutritional status of the community-dwelling older population in Bahir Dar City, Northwestern Ethiopia. Only 32.1% and 34.7% of study participants had normal nutritional status based on MNA before and after the intervention, respectively. The results are less than those of the studies in Egypt [44] and Lithuania [28], where 46% and 51.5% versus 88% and 53.7% of the studied population had normal nutrition before and after the intervention, respectively. While the nutritional status measured by BMI revealed that 40.2% and 41.6% of old age people in this study had normal weight before and after the intervention, respectively. This is more than the findings from Egypt, where 29% of participants had normal weight prior to the intervention and 34% after three months [44].

The socioeconomic disparities and differences in health literacy among the study population could be one explanation for this variance. On the other hand, the mean body weight and the BMI in this study did not change significantly, while the mean score of MNA significantly increased after the Programme. This is consistent with earlier research from Vietnam [45], Netherlands [46], Norway [47], and Finland [48], which found an increase in MNA scores but no appreciable change in body weights or BMI. This discrepancy may be due to the nature of the MNA tool, which measures many factors and is more sensitive to change than just body weight or BMI. However, the current increment might not be clinically significant that could be mainly due to the long-term nature of the nutritional outcome and the short nature of follow-up period. Hence, sustained behavioral change intervention could bring clinically meaningful changes overtime.

The overall nutritional knowledge level also increased from pre-intervention to post-intervention, consistent with earlier studies [21, 44, 49, 50]. Additionally, the mean score of the perceived susceptibility, benefits, and intentions significantly increased after the nutrition counseling compared to before the intervention. On the other hand, the mean score of perceived barriers significantly decreased after the intervention. These results are in line with the study from Iran, which found that older women's perceptions, beliefs, and behaviors around nutrition were significantly improved after HBM-based nutritional counseling [21].

Moreover, the majority of study participants had regular breakfast, lunch, and dinner practices before and after the intervention. In a similar fashion, two-thirds of older adults in the United States consumed three meals on a day of intake [51]. However, lunch was the most often skipped meal in the United States [51], in contrast to the current study, where breakfast was the meal that most study participants frequently skipped. The discrepancies might be due to cultural and health literacy differences.

Although only 2.3% of the study participants in the current study took the recommended five meals every day after the intervention, they reported changes in the number of meals and snacks they consumed per day after nutritional counseling. The results are consistent with the studies from Europe [27], Finland [48], and Vietnam [45], where study groups reported a positive change in dietary intake across all nations after receiving individualized nutritional guidance, despite the fact that participants in the various study locations had differing baseline dietary intakes.

The present study also discovered a positive effect of the implementation of the nutritional counseling program on the self-reported consumption of the majority of food categories, such as dairy products, fruits, and meat or egg intake. However, no changes were observed in the starchy staples, legumes, fats, or sweet foods. Similar findings from Egypt [49, 50] revealed statistically significant increases in the intake of dairy products, vegetables, and fruits, while statistically significant decreases in the frequency of cereal and fluid intakes were observed before and after the study intervention, but no significant changes in protein intake. Whereas, study participants in Finland reported increasing their intake of protein-rich foods after receiving individualized dietary counseling [48]. Moreover, interventional studies revealed that eating more frequent and smaller meals with protein-rich breakfast and lunch improved diet quality, lowered BMI, and preserved lean tissue mass in older adults [52, 53].

Overall, the majority of the study participants had adequate DDS and the change is also statistically significant. Yet, there was a low intake of animal products and fruits, while there was a high consumption of oils, fats, sweets, spices, coffee, and alcoholic beverages. This is comparable to the poor dietary habits of Ethiopians [54] and other African populations [50], which have a low intake of animal-source foods, vegetables, and fruits and a high intake of cereals, salt, and a rising trend in the consumption of saturated fat and oil.

Overall, to our knowledge, this is the first study that estimates the effects of behavioral model-guided nutritional counseling on community-dwelling elderly people’s nutritional status. In this study, the data collection and the counseling intervention were delivered by separate teams, which could decrease bias. However, the findings of this study should be interpreted in light of some inherent methodological limitations. First, the reliability of self-reported data and a single 24-h dietary intake recall in the study population must be considered, as well as other factors including low educational levels and the decline of sensory abilities with aging. Due to the pre-post quasi-experimental nature of the design, the study's ability to draw a causal relationship between the intervention and outcomes while controlling for extraneous confounders might be limited. Moreover, due to the variation in the characteristics of elderly, the generalizability of the study could be limited to Bahir Dar or Mahara region. The generalizability to the whole country could be limited where more context-specific and multi-centered study might be required. Related to these, the non-response rate in this study could limit the representativeness of the data. On the contrary, we have analyzed the response rate patterns and we did not get significant difference in a way that affect the outcomes of the intervention. Finally, the increment in the BMI score might not be of clinical importance that could be attributable to relatively short follow-up period making difficult to bring clinically significant improvement. However, we can understand that longer intervention period and persistent targeted health education could bring clinically meaningful results in real setting.

## Conclusion

Behavioral model-guided nutritional counseling improves the nutritional status of older people. The study participants' perception of risks, on the other hand, increased with the intervention. A participant-centered nutritional program is recommended to improve the health of community-dwelling older people. Therefore, health practitioners should train those who interact with or assist the elderly. Policymakers should integrate initiatives to educate older people about their diet and nutritional status into the federal nutrition services program already in existence. Furthermore, further research should examine the effect of more intensive counseling, considering the limitations of this study.

## Data Availability

All relevant data are within the manuscript and its Supporting Information files.

## Abbreviations

BMI: Body mass index
CI: Confidence interval
DDS: Dietary diversity score
EDHS: Ethiopian Demographic Health Survey
GNKQ: General Nutritional Knowledge Questionnaire
HBM: Health Belief Model
HFIAS: Household Food Insecurity Access Scale
IERB: Institutional Ethical Review Board
MD: Mean difference
MNA: Mini nutritional assessment
NCP: Nutrition care process
SD: Standard Deviation
TBP: Theory of Planned Behavior
TREND: Transparent Reporting of Evaluation with Nonrandomized Designs

## References

[1] HelpAge International. The State of Health and Ageing in Ethiopia: A Survey of Health Needs and Challenges of Service Provisions. Addis Ababa: HelpAge International in Ethiopia; 2013.

[2] HelpAge International (2017). Social Protection and Access to Health Services in Ethiopia, Mozambique.

[3] Audain K, Carr M, Dikmen D, Zotor F, Ellahi B (2017). Exploring the health status of older persons in Sub-Saharan Africa. Proc Nutr Soc.

[4] Kanasi E, Ayilavarapu S, Jones J (2016). The aging population: demographics and the biology of aging. Periodontol 2000.

[5] Leslie W, Hankey C (2015). Aging. Nutritional Status and Health. Healthcare.

[6] Molina-Molina E, Garruti G, Shanmugam H, Di Palo DM, Grattagliano I, Mastronuzzi T (2020). Aging and nutrition. Paving the way to better health. Pro J Intern Med..

[7] Abdu AO, Yimamu ID, Kahsay AA (2020). Predictors of malnutrition among older adults aged above 65 years in eastern Ethiopia: neglected public health concern. BMC Geriatr.

[8] Yisak H, Zemene MA, Arage G, Demelash AT, Anley DT, Ewunetei A (2023). Undernutrition and associated factors among older adults in Ethiopia: systematic review and meta-analysis. BMJ Open.

[9] HelpAge International. Needs assessment survey of older people in Kolfe Keranyio, Addis Ababa, February 2014. London: HelpAge International in Ethiopia; 2014.

[10] Hailemariam H, Singh P, Fekadu T (2016). Evaluation of mini nutrition assessment (MNA) tool among community dwelling elderly in urban community of Hawassa city. Southern Ethiopia BMC Nutr.

[11] Norman K, Haß U, Pirlich M (2021). Malnutrition in older adults—recent advances and remaining challenges. Nutrients.

[12] Sparre-Sørensen M, Kristensen GN (2016). Malnutrition related deaths. Clin Nutr ESPEN.

[13] Suma S, Furuta M, Yamashita Y, Matsushita K (2019). Aging, Mastication, and Malnutrition and Their associations with cognitive disorder: evidence from epidemiological data. Curr Oral Heal Reports.

[14] Siddiqui F, Salam RA, Lassi ZS, Das JK (2020). The intertwined relationship between malnutrition and poverty. Front Public Heal.

[15] Stockwell S, Schofield P, Fisher A, Firth J, Jackson SE, Stubbs B (2019). Digital behavior change interventions to promote physical activity and/or reduce sedentary behavior in older adults: a systematic review and meta-analysis. Exp Gerontol.

[16] Suzuki H, Kanazawa M, Komagamine Y, Iwaki M. Changes in the nutritional statuses of edentulous elderly patients after new denture fabrication with and without providing simple dietary advice. J Prosthodont Res. 2019;63(3):1–5. 10.1016/j.jpor.2018.12.010 30658908

[17] Dorner B, Friedrich EK (2018). Position of the Academy of Nutrition and Dietetics: Individualized Nutrition Approaches for Older Adults: Long-Term Care, Post-Acute Care, and Other Settings. J Acad Nutr Diet.

[18] Rigby RR, Mitchell LJ, Hamilton K, Williams LT (2020). The Use of Behavior Change Theories in Dietetics Practice in Primary Health Care: A Systematic Review of Randomized Controlled Trials. J Acad Nutr Diet.

[19] Browne S, Minozzi S, Bellisario C, Rose M, Davide S (2018). Effectiveness of interventions aimed at improving dietary behaviours among people at higher risk of or with chronic non- communicable diseases: an overview of systematic reviews. Eur J Clin Nutr.

[20] Michie S, Richardson M, Johnston M, Abraham C, Francis J, Hardeman W (2013). The behavior change technique taxonomy (v1) of 93 hierarchically clustered techniques: Building an international consensus for the reporting of behavior change interventions. Ann Behav Med.

[21] Amirzadeh Iranagh J, Motalebi SA, Mohammadi F (2018). A Theoretically Based Behavioral Nutrition Intervention for Elderly Women: A Cluster Randomized Controlled Trial. Int J Gerontol.

[22] Academy of Nutrition and Dietetics (2023). Nutrition Care Process.

[23] Kaur D, Rasane P, Singh J, Kaur S, Kumar V, Mahato DK (2019). Nutritional interventions for elderly and considerations for the development of geriatric foods. Curr Aging Sci.

[24] Mastronuzzi T, Grattagliano I (2019). Nutrition as a health determinant in elderly patients. Curr Med Chem.

[25] Siedlecki SL (2020). Quasi-Experimental Research Designs. Clin Nurse Spec.

[26] MA K, J S. Sample Size Calculators for designing clinical research: Sample size for before-after study (Paired T-test). UCSF CTSI; 2019. Available from: http://www.sample-size.net/sample-sizestudy-paired-t-test/. Cited 2019 Jul 17.

[27] Berendsen AAM, van de Rest O, Feskens EJM, Santoro A, Ostan R, Pietruszka B (1905). Changes in dietary Intake and Adherence to the NU-AGE diet following a one-year dietary intervention among European older adults—Results of the NU-AGE randomized trial. Nutrients.

[28] Spirgienė L, Damulevičienė G, Tomkevičiūtė J, Riklikienė O (2018). Nutritional status of rural community-dwelling older people and changes after following nutritional recommendations. Int J Nurs Pract.

[29] Borrelli B (2011). The assessment, monitoring, and enhancement of treatment fidelity in public health clinical trials. J Public Health Dent.

[30] Central Statistical Agency (CSA) [Ethiopia] and ICF (2016). Ethiopia Demographic and Health Survey 2016. Central Statistical Agency.

[31] Coates J, Swindale A, Bilinsky P (2007). Household Food Insecurity Access Scale (HFIAS) for Measurement of Food Access: Indicator Guide (V.3).

[32] Asesefa Kisi M, Tamiru D, Teshome MS, Tamiru M, Feyissa GT (2018). Household food insecurity and coping strategies among pensioners in Jimma Town. South West Ethiopia BMC Public Health.

[33] Endale W, Mengesha ZB, Atinafu A, Adane AA (2014). Food Insecurity in Farta District, Northwest Ethiopia: a community based cross – sectional study. BMC Res Notes.

[34] Tsegaye AT, Tariku A, Worku AG, Abebe SM, Yitayal M, Awoke T (2018). Reducing amount and frequency of meal as a major coping strategy for food insecurity. Arch Public Heal.

[35] Ty H, Krawinkel M (2016). Dietary Diversity Score: A Measure of Nutritional Adequacy or an Indicator of Healthy Diet?. J Nutr Heal Sci.

[36] Cokieng CBC, Gutierrez LAR, Manaloto ANP, See JPC, Tan JHC, Bullecer ER (2014). Validity of dietary diversity score as an indicator of nutrient adequacy among older adults in pasay city, philippines. Acta Med Philipp.

[37] Christiansen TB, Lauritsen JM. EpiData - Comprehensive Data Management and Basic Statistical Analysis System. Odense Denmark: EpiData Association; 2010. Available from:http://www.epidata.dk. Cited 2019 Nov 8.

[38] Corp IBM (2015). IBM SPSS Statistics for Windows, Version 23.0.

[39] Pan-African Clinical Trials Registry; https://www.who.int/clinical-trials-registry-platform/network/primary-registries/pan-african-clinical-trials-registry-pactr

[40] Jarlais DC DES, Lyles C, Crepaz N, Group the T (2004). Improving the Reporting Quality of Nonrandomized Evaluations of Behavioral and Public Health Interventions: The TREND Statement. Am J Public Health..

[41] Nestle Nutrition Institute (2011). Nutrition screening - a guide to completing the Mini Nutritional Assessment (MNA).

[42] Cashin K, Oot L (2018). Guide to Anthropometry: A Practical Tool for Program Planners, Managers, and Implementers.

[43] World Medical Association (2013). WMA Declaration of Helsinki- Ethical principles for Medical Research Involving Human Subjects. Bull World Health Organ.

[44] Abdelwahed AY, Mhmoud M, Algameel M, Tayel DI (2018). Effect of a Nutritional Education Program on Nutritional Status of Elderly in Rural Areas of Damanhur City. Egypt Int J Nurs Sci.

[45] Thanh H, Mph N, Pavey TG, Collins PF, Nguyen NV, Pham TD, et al. Effectiveness of Tailored Dietary Counseling in in Treating Malnourished Outpatients with Chronic Obstructive Pulmonary Disease: A Randomized Controlled Trial. J Acad Nutr Diet. 2019;120(5):1–15. 10.1016/j.jand.2019.09.013 31786177

[46] Twisk JWRR, Bosmans JE, van der Pols-Vijlbrief R, Wijnhoven HAHH, Visser M, Van Der Pols-vijlbrief R (2017). Targeting the underlying causes of undernutrition. Cost-effectiveness of a multifactorial personalized intervention in community-dwelling older adults: A randomized controlled trial. Clin Nutr..

[47] Andersson J, Hulander E, Rothenberg E, Iversen PO (2017). Effect on Body Weight, Quality of Life, and Appetite Following Individualized, Nutritional Counselling to Home-Living Elderly After Rehabilitation – An Open Randomized Trial. J Nutr Heal Aging.

[48] Nykänen I, Rissanen TH, Sulkava R, Hartikainen S (2013). Effects of individual dietary counseling as part of a Comprehensive Geriatric Assessment (CGA) on nutritional status: A population-based intervention study. J Nutr Heal Aging.

[49] Shalaby S, MM A, El Dean H, Mohamed RA, Neelima Gupta A, Singh R (2016). Impact of nutritional health educational program `on elderly persons’ nutritional knowledge , attitude and practice. RJPBCS.

[50] Mohamed RA, Awad MM, Shalaby SI, Abdelsatar HN (2013). Effect of Nutritional Health Education Program on Elderly Nutritional Knowledge, Attitude and Practice in Abu Khalifa Primary Health Care Center. Ismailia Governorate Med J Cairo Univ.

[51] Krok-Schoen JL, Jonnalagadda SS, Luo M, Kelly OJ, Taylor CA (2019). Nutrient intakes from meals and snacks differ with age in middle-aged and older americans. Nutrients.

[52] Aljuraiban GS, Chan Q, Oude Griep LM, Brown IJ, Daviglus ML, Stamler J (2015). The Impact of Eating Frequency and Time of Intake on Nutrient Quality and Body Mass Index: The INTERMAP Study, a Population-Based Study. J Acad Nutr Diet.

[53] Norton C, Toomey C, McCormack WG, Francis P, Saunders J, Kerin E (2016). Protein supplementation at breakfast and lunch for 24 weeks beyond habitual intakes increases whole-body lean tissue mass in healthy older adults. JNutr.

[54] Shiferaw F, Letebo M, Misganaw A, Feleke Y, Gelibo T (2018). Non-communicable Diseases in Ethiopia: Disease burden, gaps in health care delivery and strategic directions. Ethiop J Heal Dev.

